# Development of Transgenic Papaya through *Agrobacterium*-Mediated Transformation

**DOI:** 10.1155/2013/235487

**Published:** 2013-08-28

**Authors:** Md. Abul Kalam Azad, Md. Golam Rabbani, Latifah Amin, Nik Marzuki Sidik

**Affiliations:** ^1^Centre for General Studies, Universiti Kebangsaan Malaysia, 43600 UKM Bangi, Selangor, Malaysia; ^2^Department of Agricultural Extension, Khamarbari, Farmgate, Dhaka 1215, Bangladesh; ^3^Department of Horticulture, Bangladesh Agricultural University, Mymensingh 2202, Bangladesh; ^4^Faculty of Science and Technology, Universiti Kebangsaan Malaysia, 43600 UKM Bangi, Selangor, Malaysia

## Abstract

Transgenic papaya plants were regenerated from hypocotyls and immature zygotic embryo after cocultivation with *Agrobacterium tumefaciens* LBA-4404 carrying a binary plasmid vector system containing neomycin phosphotransferase (*nptII*) gene as the selectable marker and *β*-glucuronidase (GUS) as the reporter gene. The explants were co-cultivated with *Agrobacterium tumefaciens* on regeneration medium containing 500 mg/L carbenicillin + 200 mg/L cefotaxime for one week. The cocultivated explants were transferred into the final selection medium containing 500 mg/L carbenicillin + 200 mg/L cefotaxime + 50 mg/L kanamycin for callus induction as well as plant regeneration. The callus derived from the hypocotyls of *Carica papaya* cv. Shahi showed the highest positive GUS activities compared to *Carica papaya* cv. Ranchi. The transformed callus grew vigorously and formed embryos followed by transgenic plantlets successfully. The result of this study showed that the hypocotyls of *C. papaya* cv. Shahi and *C. papaya* cv. Ranchi are better explants for genetic transformation compared to immature embryos. The transformed *C. papaya* cv. Shahi also showed the maximum number of plant regeneration compared to that of *C. papaya* cv. Ranchi.

## 1. Introduction

Papaya (*Carica papaya* L.) is a tropical fruit having commercial importance because of its high nutritive and medicinal value [1]. It is also a good source of vitamins A and C and the proteolytic enzyme papain and chymopapain [2, 3]. The origins of papaya are south Mexico and Costa Rica [4]. Major papaya cultivating countries are India, Brazil, Mexico, Nigeria, Indonesia, China, Peru, Thailand, and Philippines [5]. Papaya cultivation has been affected by a number of diseases especially those caused by the papaya ringspot virus (PRSV) [6, 7], papaya bunchy top bacterium [8], and the fungus *Phytophthora palmivora* [9]. Traditional breeding of papaya cultivars for resistance to these maladies had limited success. The rapid development in biotechnology, especially genetic transformation of plants, has made it possible to introduce selected genes into plants to control plant diseases and pests. Crop improvement to solve disease problems of a tree species like papaya has been enhanced by gene transfer techniques [10]. 

 Genetic engineering of plants has evoked great interest in developing modern technology for crop improvement. Many transgenic plants have been successfully produced with remarkable results such as resistance to chemicals, pests, and diseases [11]. The success of any genetic transformation protocol depends on three steps such as reproducible shoot regeneration system, establishment of an inoculation system, and the method of transformed shoot selection [12]. *Agrobacterium-*mediated transformation protocols differ from one plant species to another, within species and from one cultivar to another [13]. *Agrobacterium-*mediated transformation has remarkable advantages over direct transformation method as it reduces the copy number of transgene, potentially leading to fewer constructs with transgene cosuppression and instability [14]. *Agrobacterium-*mediated transformation is a single-cell transformation system which has been used more frequently compared to direct transformation [15].

 Genetic transformation of papaya using *Agrobacterium *or microprojectile bombardment has been reported by scientists [16–18]. Fitch et al. [16] developed transgenic papaya plants from somatic embryos after cocultivation with *A. tumefaciens* strain C58-Z707. Fitch and Manshardt [19] transformed and regenerated transgenic plants using microprojectile bombarded from immature zygotic embryos and hypocotyls of Sunrise Solo and Kapoho Solo. Manshardt [2] reported that papaya mosaic virus resistant variety can be developed through genetic transformation system using viral Coat Protein (CP) gene. Pang and Sanford [20] transformed papaya leaf discs, stems, and petioles of Sunrise Solo and Kapoho with *A. tumefaciens* strains GV3111, but they could not regenerate plantlets from these calli. Tennant et al. [21] reported that the transgenic papaya expressing the coat protein gene showed high levels of resistance against the severe papaya ringspot virus in Hawaii. Type of explants and genotype are important factors for successful genetic transformation using *A. tumefaciens* of papaya.

*C. papaya *cv. Shahi and *C. papaya *cv. Ranchi are two popular local papaya varieties which are widely grown and contribute to major sources of income for the farmers in Bangladesh. The first is a native variety, while *C. papaya *cv. Ranchi was first introduced in India. Cultivation of these varieties was affected by many diseases caused by various pathogens and viruses. Past research [22] has shown that conventional technology such as breeding could not solve these problems, so the best alternative is to develop disease-resistant varieties through genetic engineering. In order to transfer disease-resistant genes into these two varieties, suitable and efficient genetic transformation systems have to be established for the two varieties. To date, there has been no attempt on the development of genetic transformation through *A. tumefaciens* using hypocotyls and immature zygotic embryos of these two papaya varieties. More regenerable tissues and cells are available in hypocotyls and immature zygotic embryos of papaya. Therefore, the present study has been undertaken to develop the protocol for genetic transformation using hypocotyls and immature zygotic embryos of *C. papaya* cv. Shahi and *C. papaya* cv. Ranchi through *A. tumefaciens* LBA-4404.

## 2. Materials and Methods

### 2.1. Plant Materials

*Carica papaya *cv. Shahi and *Carica papaya *cv. Ranchi are dioecious local varieties. The first is a native species which was released in Bangladesh in 1992 by the Bangladesh Agricultural Research Institute, Joydebpur, Gazipur, Bangladesh while *C. papaya* cv. Ranchi was first introduced in India. The mature seeds and 120-days-old fresh fruits of *C. papaya *cv. Shahi and *C. papaya *cv. Ranchi were washed with detergent and tap water several times. The mature seeds were disinfected with 0.2% HgCl_2_ for 10 minutes followed by five times rinse with sterilized distilled water. The mature seeds were germinated, and hypocotyls from 10-days-old seedlings were used for inoculation. The immature zygotic embryos were separated from 120-days-old fruits and sliced for inoculation.

### 2.2. Culture Medium for Regeneration

The regeneration medium was half strength MS salt supplemented with 5 mL nicotinic acid, 1.0 mg pyridoxine-HCl, 100 mg myo-inositol, 1.0 mg NAA, 0.5 mg kinetin, 160.0 mg adenine sulphate, 1.0 mg GA_3_, 1.0 g casein hydrolysate, and 30 g sucrose per litre. The Luria-Bertani (LB) medium was 15 g NaCl, 10 g peptone, 5 g yeast extract, and 15 g agar per litre. 

### 2.3. Transformation Agent

*A. tumefaciens* strain LBA-4404 carrying a binary plasmid vector system containing neomycin phosphotransferase (*nptII) *with GUS and kanamycin-resistant genes.

### 2.4. Transformation Protocol

A single colony of *A. tumefaciens* was inoculated in 15 mL liquid LB medium containing 50 mg/L kanamycin and grown at 28°C on a gyratory shaker (180 rpm). Hypocotyls and immature zygotic embryos were immersed in *A. tumefaciens *LBA-4404 for one minute under gentle agitation. The explants were blotted on sterile filter paper and cocultivated with *A. tumefaciens* on regeneration medium for 6 hours. Cocultivated explants were transferred into regeneration medium containing 500 mg/L carbenicillin and 200 mg/L cefotaxime for one week. After one week, the explants were transferred to regeneration medium containing 200 mg/L Cefotaxime + 50 mg/L kanamycin for callus induction.

### 2.5. GUS Staining

*β*-glucuronidase activity was assayed histochemically using a small amount of callus [23]. The callus was stained by placing the tissue in 1 mL X-gluc staining phosphate buffer in a small vial. The sample was incubated at 37°C overnight and examined under a light microscope for the evidence of blue colour in the transformed sample.

### 2.6. Acclimatization of Plantlets

 Well-developed plantlets were removed from the culture and washed in sterilized water to remove traces of the medium. These plantlets were transplanted into plastic pots containing a mixture of autoclaved cocopeat, sand, and garden soil (1 : 1 : 1). The plants were grown in growth chambers (24 ± 2°C and 16 h photoperiod with 80% relative humidity) and were sprayed with Hoagland solution once a week. After two weeks, the plants were transferred to the greenhouse. Thirty-six percent of the plantlets have been successfully transferred into the pot. 

### 2.7. Data Collection and Analysis

 The complete randomized design was used for all experiments. For each treatment, 30 explants were cultured (6 explants per culture tube and 3 replicates per treatment), and the experiment was repeated three times. The cultures were observed periodically and morphological changes were recorded at regular intervals. Data were recorded and analyzed using SAS statistical package. Significant differences were assessed by using Duncan's Multiple Range Test (DMRT) at the 5% probability level [24]. The results were expressed as mean ± standard error (SE).

## 3. Results and Discussion

 Results of genetic transformation of *C. papaya* cv. Shahi and *C. papaya* cv. Ranchi are presented in Table 1. The hypocotyls and immature zygotic embryos of *C. papaya* cv. Shahi and *C. papaya* cv. Ranchi were placed into regeneration medium for cocultivation after the infection with *A. tumefaciens *strains LBA-4404. The cocultivated explants were transferred into regeneration medium containing 500 mg/L carbenicillin + 200 mg/L cefotaxime after one week. The explants became swollen and the bacteria engulfed some of the explants during the cocultivation with *A. tumefaciens*. Then the explants were transferred into the final selection medium containing 500 mg/L carbenicillin + 200 mg/L cefotaxime + 50 mg/L kanamycin and incubated for 3 weeks. The hypocotyls of *C. papaya* cv. Shahi showed the maximum response to acclimatization while the lowest response was found in immature zygotic embryos of *C. papaya* cv. Ranchi (Table 1). Upon subculturing on the same medium, the explants infected by *A. tumefaciens* gradually increased in size to form callus. The hypocotyls of *C. papaya* cv. Shahi induced maximum number of callus compared to immature zygotic embryos and callus induction was found to be higher in* C. papaya* cv. Shahi compared to *C. papaya* cv. Ranchi. This might be due to the fact that hypocotyls were more juvenile and therefore more suitable for callus induction after genetic transformation. Genotype also plays an important part for genetic transformation of *Carica papaya.* de la Riva et al. [13] reported that *Agrobacterium*-mediated transformation protocols differ from one species and cultivar to another. Fitch et al. [17] has also successfully developed transgenic papaya plants using *Agrobacterium-*mediated transformation using hypocotyl section of *Carica papaya* cv. Kapoho.

### 3.1. Selection of Kanamycin-Resistant Transformants

The transformed and nontransformed calli were cultured onto medium containing 50 mg/L kanamycin. All transformed callus grew vigorously in this medium and formed embryos followed by plantlets formation. But the nontransformed callus did not grow and turned black in this medium. This result indicated that the kanamycin resistant genes inserted in the explants gave the putative positive result which was confirmed by GUS expression. Ficth et al. [17] reported that the hypocotyls of papaya cocultivated with *Agrobacterium tumefaciens* containing binary cosmid vectors pGA482GG showed positive results. Pang and Sanford [20] also reported similar findings when the petiole explants of papaya were cocultivated with *A. tumefaciens* containing LBA-4404. Ying et al. [22] reported that kanamycin resistant papaya plants were produced from somatic embryos after cocultivation with *A. tumefaciens* containing LBA-4404. 

### 3.2. GUS Expression

**Figure 1:**
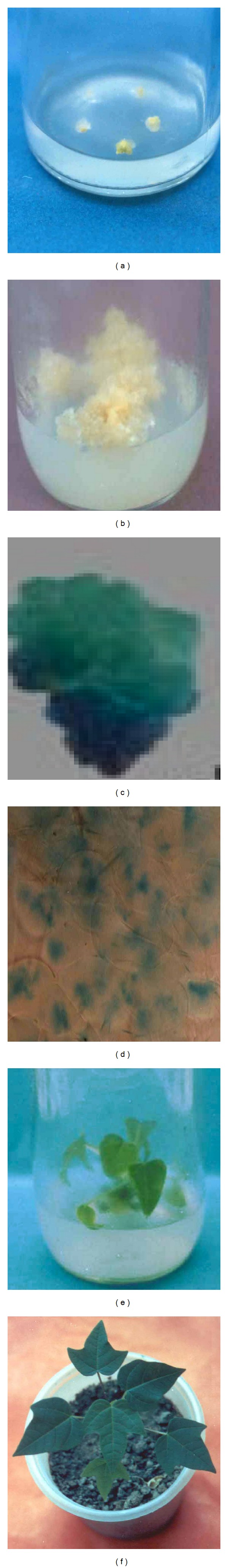
Genetic transformation of hypocotyls of *C. papaya* cv. Shahi. (a) Callus formation after infection by *Agrobacterium tumefaciens* strains LBA-4404. (b) Embryogenic callus after infection by *Agrobacterium tumefaciens* strains LBA-4404. (c) Transgenic expression indicating blue colour on callus. (d) Blue colour showing the transgenic expression in cross section of transformed callus. (e) Transgenic plant in culture tube. (f) Acclimatization to ex-vitro condition of transgenic plant.

Some of the transformed callus and nontransformed callus were stained histochemically for the detection of GUS expression. Blue colour indicated that the transformation of GUS gene was positive under the stereo-microscope (Figure 1). The callus of *C. papaya* cv. Shahi showed the highest GUS activity compared to *C. papaya* cv. Ranchi. The control callus was not stained blue during the study. Similar result was reported by Ficth et al. [17] for hypocotyls of papaya cv. Kapoho cocultivated with *A. tumefaciens* containing binary cosmid vectors pGA482GG. The hypocotyls of *C. papaya* cv. Shahi induced maximum number of callus which was statistically identical with hypocotyls of *C. papaya* cv. Ranchi while the lowest number of callus was found in immature zygotic embryo of *C. papaya* cv. Ranchi which was statistically identical with immature zygotic embryo of *C. papaya* cv. Shahi. On the other hand, the maximum number of regenerated plants was recorded in hypocotyls of *C. papaya* cv. Shahi and the lowest number of plant regeneration was found in immature zygotic embryo of *C. papaya* cv. Ranchi.

## 4. Conclusion

 The results of this study confirmed that the hypocotyls of *C. papaya* cv. Shahi and *C. papaya* cv. Ranchi are better explants for genetic transformation compared to immature embryos. The transformed *C. papaya* cv. Shahi also showed maximum number of plant regeneration compared to *C. papaya* cv. Ranchi. Our study indicated that the hypocotyls of *C. papaya* cv. Shahi can be used for the successful development of transgenic papaya. This result will be useful for academicians and plant breeders for the development of transgenic papaya variety. 

## Figures and Tables

**Table 1:** Transformation efficiency and GUS activities of hypocotyls and immature zygotic embryos of *Carica papaya* cv. Shahi and *Carica papaya* cv. Ranchi infected by *Agrobacterium tumefaciens* strains LBA-4404.

Treatment	Survivability (%) (Mean ± SE)	Callus formation (%) (Mean ± SE)	No. of GUS positive response (%) (Mean ± SE)	No. of somatic embryos produced/explants (Mean ± SE)	No. of regenerated plantlets/explants (Mean ± SE)
*C. papaya* cv. Shahi					
Hypocotyls	6.83 ± 0.26	42.41 ± 2.72	27.48 ± 1.40	8.41 ± 0.65	5.36 ± 0.47
Zygotic embryos	5.50 ± 0.21	34.46 ± 1.12	20.06 ± 0.93	6.53 ± 0.60	3.38 ± 0.60
*C. papaya* cv. Ranchi					
Hypocotyls	4.73 ± 0.32	38.80 ± 0.79	22.80 ± 1.50	7.60 ± 0.49	4.06 ± 0.35
Zygotic embryos	4.50 ± 0.15	28.90 ± 1.64	28.90 ± 1.64	4.43 ± 0.45	2.11 ± 0.17
